# Intestinal Flora: A Potential New Regulator of Cardiovascular Disease

**DOI:** 10.14336/AD.2021.1022

**Published:** 2022-06-01

**Authors:** Yifei Zou, Xianjing Song, Ning Liu, Wei Sun, Bin Liu

**Affiliations:** Department of Cardiology, The Second Hospital of Jilin University, Changchun, China

**Keywords:** Intestinal flora, cardiovascular disease, diagnosis, therapy

## Abstract

Although substantial progress has been made in reducing the burden of the disease by preventing the risk factors of cardiovascular disease (CVD), potential risk factors still exist and lead to its progression. In recent years, numerous studies have revealed that intestinal flora can interfere with the physiological processes of the host through changes in composition and function or related metabolites. Intestinal flora thus affects the occurrence and development of a variety of CVDs, including atherosclerosis, ischemic heart disease, and heart failure. Moreover, studies have found that interventions for intestinal flora and its metabolites provide new opportunities for CVD treatment. This article mainly discusses the interaction between the human intestinal flora and its metabolites, the occurrence and development of CVD, and the potential of intestinal flora as a new target for the diagnosis and treatment of CVD.

The epidemiological transition of the 20th century is mainly reflected in the decrease in the number of deaths caused by infectious diseases and the increase in the number of deaths caused by non-communicable diseases. Among non-communicable diseases, cardiovascular disease (CVD) is the leading cause of death worldwide [1]. CVD mainly includes ischemic heart disease (IHD), heart failure (HF), and peripheral artery disease, and its incidence increases worldwide every year. According to the latest data from the World Health Organization, 17.9 million people died of CVD in 2019, accounting for 32% of global deaths. Of these deaths, 85% were due to heart attacks and strokes. Of the 17 million premature deaths (under 70) caused by non-communicable diseases in 2019, 38% were caused by CVD. The recognized risk factors for CVD include hyperlipidemia, hypertension (HTN), diabetes, smoking, and lack of physical exercise [2]. Although the mortality rate of CVD is high, the prevention of risk factors can reduce the prevalence of CVD.

The interaction between flora inside the gut and its host has drawn a lot of attention in recent years. Furthermore, increasing evidence indicates that an imbalance in intestinal flora may be a potential risk factor for CVD. Moreover, the imbalance in intestinal flora may also be related to diseases, such as inflammatory bowel disease, neurological diseases, and allergies [3-5]. Numerous studies have confirmed a close relationship between intestinal flora and CVD. First, changes in the composition and function of intestinal flora associated with diseases are related to atherosclerosis (AS), IHD, HF, and type 2 diabetes [6, 7]. Second, metabolites of intestinal flora have been identified as an important factor in the development of CVD [8]. Intestinal flora can produce biologically active metabolites, which can affect the physiological function of the host [9]. Through the study of transplanted intestinal flora, specific intestinal flora-dependent pathways and downstream metabolites can lead to the occurrence of CVD through specific host receptors and have shown significant clinical relevance in human studies [10]. In this review, we emphasized the role of intestinal flora and its metabolites in the occurrence and development of CVD, and the potential of intestinal flora as new predictors and therapeutic targets of CVD.

## 1. Intestinal flora and its metabolites

Trillions of microbial cells exist throughout the human body and play an important role in human health and disease. This type of microbial cell population has the highest density in the intestine, where they together form a complex microbial community called intestinal flora. Intestinal flora mainly includes archaea, bacteria, and eukaryotes. They establish a complex nutritional relationship from symbiosis to parasitism with each other and with the human host [11]. The types of bacteria found in the human gut microbiome mainly include three phyla: *Bacteroidetes*, *Firmicutes*, and *Actinobacteria*. According to reports, the number and composition of bacteria vary in different parts of the gastrointestinal tract. There are fewer bacteria in the upper part of the stomach and the small intestine. Furthermore, the number of bacteria from the jejunum to the colon gradually increases. Moreover, intestinal flora is 10 times more abundant than somatic and germ cells, and its collective genome is 150 times larger than the human genome. In a human body, 150-170 species of bacteria dominate and play a variety of functions, such as regulating body metabolism, neurodevelopment, and energy homeostasis [12].

Under normal conditions, intestinal flora can regulate human physiology by releasing different metabolites. However, an imbalance in intestinal flora reduces beneficial substances, including short-chain fatty acids (SCFA), leading to the destruction of the intestinal barrier. It also leads to the production of numerous toxic substances, such as lipopolysaccharide (LPS), trimethylamine-N-oxide (TMAO), and phenylacetyl glutamine (PAGln), which enter the blood and ultimately accelerate the progression of CVD [13].

### 1.1 Lipopolysaccharide

LPS is a structural compound found in the outer membrane of gram-negative bacteria. It is comprised of a hydrophilic polysaccharide and a hydrophobic component called lipid A, which is responsible for the main biological activities of LPS and is recognized as pathogen-related molecules by immune cells [14]. LPS can be recognized by Toll-like receptor 4 (TLR4), which induces inflammation. The receptor is expressed on immune cells (such as macrophages) and many other types of cells (including liver and fat cells). Under normal circumstances, the intestinal epithelium acts as a barrier to prevent LPS from shifting. However, under the conditions of ischemia and intake of high fat foods, the intestinal barrier function may be destroyed, leading to LPS translocation. Thus, the LPS level in blood increases, causing systemic inflammation, which leads to a variety of diseases, including dyslipidemia, insulin resistance, and CVD [15].

The possible mechanism of LPS-induced CVD is as follows. First, LPS upregulates inflammation-related genes and significantly increases the plasma concentrations of tumor necrosis factor α (TNFα) and interleukin 6 (IL-6) [16]. This inhibits the oxidation of mitochondrial fatty acids in cardiomyocytes. As the heart mainly produces adenosine triphosphate through the oxidation of fatty acids [17], this will eventually cause the mechanical function of the heart to decline [18]. Second, LPS stimulates macrophages to release pro-atherosclerotic cell inflammatory factors (such as TNFα, IL-1, IL-6, IL-8, and IL-12) to accelerate the progression of AS [19] and enhances platelet aggregation through TLR4-mediated leukocyte cathepsin G activation [7]. In vitro experiments have shown that TLR4 inhibitors can attenuate this effect [20]. Simultaneously, LPS can participate in the process of thrombosis by upregulating the expression of macrophage tissue factor. LPS can also enhance the platelet-mediated monocyte production of pro-inflammatory cytokines under the action of common agonists such as Pam3CSK4 when interacting with TLR4 to participate in the process of thrombosis [21-23]; therefore, LPS can induce inflammation in the body through a variety of ways and increase platelet activation and thrombosis, thereby accelerating the development of a variety of CVDs. In-depth research on LPS is conducive to the better treatment of diseases.

### 1.2 Trimethylamine-N-oxide

Studies have shown that choline and L-carnitine in the diet can be metabolized by intestinal flora into trimethylamine (TMA), which when absorbed reaches the liver through portal vein circulation and is then rapidly oxidized to TMAO by flavin-containing monooxygenases (FMO) in the liver [24]. Increasing evidence shows that the microbiota-dependent metabolite TMAO is related to CVD [25-29]. Pro-CVD effects of intestinal flora-dependent TMAO include inhibition of cholesterol reverse transport, promotion of increased cholesterol accumulation in macrophages and formation of foam cells, pro-inflammatory changes in arterial vessel walls, induction of platelet hyperresponsiveness, and increased potential for arterial thrombosis [30]. In clinical studies on stroke patients [31], it was found that the increase in the TMAO level is dose-dependent with the risk of recurrent stroke and secondary cardiovascular events, which may be related to the increase in pro-inflammatory monocytes caused by TMAO. In addition, TMAO is associated with chronic kidney disease [32] and Alzheimer's disease [33].

Numerous studies in recent years have identified interesting issues that require further exploration. Platelet hyperresponsiveness mediated by increased TMAO levels can be reduced by low-dose aspirin. Some experts believe that increased levels of the metabolite TMAO produced by gut microbes may also weaken the antiplatelet effect of low-dose aspirin [34]. This brings us to the question: is low-dose aspirin beneficial for high-risk CVD patients with elevated TMAO? Furthermore, TMAO synthesis depends on microbial metabolism to produce TMA, which is mediated by the expression of choline TMA lyase from the *CUTC* gene of intestinal flora. Increased *CUTC* gene expression can increase the host's platelet reactivity and thrombosis potential by increasing the level of TMAO. Thus, we can speculate that inhibiting the microbial *CUTC* gene may be a promising treatment to inhibit the development of the disease by reducing the possibility of thrombosis [35]. Finally, as the main member of the FMO enzyme family, FMO3 expression levels showed a significant difference between genders (women showed 1,000 times higher expression levels than men), and a significant positive correlation between the FMO3 expression in the liver and AS lesions was found [36]. Among all human FMOs tested, only the expression of liver FMO3 was positively correlated with plasma TMAO levels [37]. In the body, the regulation of FMO3 can directly affect the systemic TMAO level and participate in the changes of platelet reactivity and thrombosis potential that are diet-dependent and intestinal flora-dependent [38]. Therefore, future research on human TMAO and CVD-related mechanisms may lead to unexpected gains in the activity of FMO3.

### 1.3 Phenylacetyl glutamine

After the essential amino acid phenylalanine is ingested, most of it is absorbed by the small intestine. Unabsorbed phenylalanine can be metabolized by intestinal flora to form phenylpyruvate, which then forms phenylacetic acid [39]. After phenylacetic acid is absorbed into the portal vein, it is metabolized in the liver and conjugated with glutamine (Gln) or glycine (Gly) to produce PAGln or PAGly, respectively [40]. There is a clinical association between elevated PAGln levels and CVD risk [41, 42]. Large-scale clinical studies have found that, compared with that in patients without major adverse cardiovascular events (MACE), PAGln plasma levels are significantly higher in patients with MACE and can be used as an independent predictor of MACE [43].

Beta-blockers have many clinical benefits in some patients with CVD [44]. By treating mice with widely used β-blockers in clinical practice, the hyperresponsiveness of platelets caused by PAGln and the acceleration of thrombosis in the body can be reduced, and the thrombosis-promoting effect of PAGln can be reversed [43]. Current studies suggest that some of the clinical benefits observed with beta-blocker therapy may be mediated in part by attenuating PAGln-triggered adrenergic receptor (ADR) signal transduction in the body. PAGln represents a new intestinal flora-dependent metabolite that promotes the progression of CVD and has broad research prospects. However, the current research is limited to the interaction between PAGln and ADR, and its physiological effects and clinical applications still depend on further exploration and discovery.

### 1.4 SCFA

SCFA are derived from the fermentation of dietary fiber in the colon by intestinal flora. Acetate, propionate, and butyrate account for 90% of SCFA produced by the intestinal microbiota [45]. They play an important role in the host's energy metabolism, fat and cholesterol synthesis, and gluconeogenesis. Acetate is found at higher levels in the body, whereas propionate and butyrate have lower levels. This is because butyrate is the first energy source for colon cells and is locally consumed, whereas propionate is metabolized by the liver after being discharged into the portal vein. Acetate becomes the most abundant SCFA in the peripheral circulation.

The current research on SCFA is still very limited and cannot explain its protective role in the occurrence and development of CVD. The possible mechanisms are as follows. SCFA affect gene transcription by inhibiting histone deacetylase activity. SCFA can also regulate cell function by interacting with receptors on target cells. Studies have found that SCFA have anti-inflammatory properties and can inhibit nuclear factor kappa-B (NF-κB) activity in immune cells, resulting in a reduction in the production of pro-inflammatory cytokines, including interferon γ (IFN-γ), IL-1β, and IL-2. However, the specific mechanism still depends on further experimental research.

SCFA receptors are G-protein-coupled receptors (GPRs), with seven receptors confirmed to date: GPR41, GPR42, GPR43, GPR91, GPR109A, GPR164, and vascular olfactory receptor 78 (OLFR78) [46, 47]. SCFA can activate GPR43 and GPR109A to reduce pathological myocardial remodeling caused by hypertension [48]. By activating GPR43 or GPR109A, SCFA can also inhibit the expression of genes encoding inflammatory cytokines, chemokines, and pro-fibrotic proteins in the kidneys of diabetic mice, therefore, the mice show less proteinuria and glomerular hypertrophy. Further, podocyte damage and interstitial fibrosis reduce the incidence of diabetic nephropathy, playing a role in kidney protection [49]. In addition, SCFA can induce hyperpolarization of the intestinal epithelial cell membrane in a GPR43-dependent manner and activate the NLRP3 inflammasome in the intestinal epithelium. SCFA also activate GPR109A to stimulate the production of IL-18, promoting intestinal homeostasis and preventing colitis [50]. GPR43-deficient mice showed increased sensitivity to dextran sodium sulfate-induced colitis [51]. In recent studies, SCFA have been shown not only to exert its anti-inflammatory and protective properties in the intestines but also to reach the circulatory system, directly affecting the adipose tissue, brain, and liver and producing beneficial metabolic effects [52]. SCFA exert these properties in a variety of ways, but the underlying mechanism is still unclear.

SCFA are becoming an emerging therapeutic target for systemic inflammation and metabolic diseases, including CVD [53, 54], and the future development of CVD therapeutic interventions for SCFA is promising.

## 2. Intestinal flora and cardiovascular disease

### 2.1 Atherosclerosis

AS is the main underlying cause of CVD. AS is characterized by the formation of lipid-rich plaques in large and medium-sized blood vessels and is considered a chronic inflammatory disease of the arterial vessel wall. Chronic inflammation and dyslipidemia are considered important factors that lead to AS. Recent studies have found that an imbalance in intestinal flora is also a key pathogenic factor for AS. Yoshida et al. [55] found an abundance of *Bacteroides vulgatus* and *Bacteroides dorei* in the intestinal flora of patients with AS. They are the dominant species of *Bacteroides* in the human intestinal flora.

Previous studies have found that AS is affected by the innate and adaptive immune systems, and cytokines are involved in all stages of AS formation [56]. LPS can cause systemic inflammation, helping to destroy innate and acquired immune responses and accelerating the progression of AS [57]. Clinical studies have shown that patients with elevated serum LPS levels have an increased risk of AS [58]. In a study on apolipoprotein E knockout (ApoE-/-) mice [55], it was found that the plasma levels of LPS in mice treated with *Bacteroides* were significantly reduced, and the plasma levels of atherogenic cell inflammatory factors, such as IL-2 and 4, were lower than untreated control group.

In further research on the function of the intestinal flora, we found two interesting phenomena. First, the gene expression levels of LpxA and LpxD, essential acyltransferases involved in lipid A biosynthesis, were significantly lower than those of the control group. This may explain the decrease in LPS of mice treated with *Bacteroides*. Second, the mRNA expression of the tight junction gene *Zo1* in *Bacteroides*-treated mice was significantly increased, leading to a decrease in intestinal permeability and inhibiting ectopic LPS from entering the circulatory system to cause inflammation in the body. Gavage of *B. vulgatus* and *B. dorei* can reduce the production of LPS in intestinal flora, reducing the plasma level of LPS and effectively inhibiting the pro-inflammatory immune response, which in turn significantly reduces the formation of AS lesions in mice. However, in a study on ApoE-/- mice, Kasahara et al. [59] found that, compared with conventionally fed mice, aseptically fed mice showed a significant increase in plasma cholesterol levels due to the lack of intestinal flora. However, unexpectedly, the area of ??AS plaques was significantly reduced. Further research found that the pro-inflammatory cytokines in the macrophages and the aorta of the mice fed a sterile diet decreased significantly, which may be related to the decrease in circulating LPS levels and the weakening of the secondary inflammatory response. These studies have shown that the lack of intestinal flora can reduce inflammatory cytokines and chemokines in systemic circulation, inhibiting AS.

The intestinal flora of caspase1 knockout (Casp1-/-) mice can promote an increase in white blood cell levels and accelerate the accumulation of pro-inflammatory cytokines and neutrophils in AS plaques, leading to the progression of AS. Transplanting fecal intestinal flora from Casp1^-/-^ mice to low-density lipoprotein receptor knockout (Ldlr^-/-^) mice can lead to increases in AS plaques in the aortic roots, number of blood leukocytes (particularly monocytes and neutrophils), pro-inflammatory cytokines (IFN-γ, IL-2, and IL-1β), and neutrophil accumulation in AS plaques, and reduced SCFA levels in the cecum [60]. These results indicate that there is a causal relationship between intestinal flora and AS through inflammatory cells and inflammatory factors. Therefore, studies have shown that changes in the composition and function of intestinal flora have a great impact on the occurrence and development of AS, but whether there is an inevitable causal link between them is worthy of in-depth study. The rational application of probiotics is still worth exploring, as the therapeutic value for improving the systemic inflammatory response and inhibiting the progression of AS is immense.

### 2.2 Ischemic heart disease

Increasing evidence shows that metabolites of intestinal flora are related to the development of IHD. TMAO has received extensive attention because of its potential role as a risk factor for IHD. Several studies have shown that circulating TMAO is a marker of coronary atherosclerotic burden, and there is a dose-dependent relationship between its level and atherosclerotic burden. Elevated TMAO levels have been positively correlated with the carotid plaque burden [61, 62]. TMAO is also a powerful predictor of cardiovascular risk for diabetic patients [63]. Heianza et al. [64] found that long-term changes in plasma TMAO levels were significantly related to the incidence of IHD in healthy women. Women with chronically high levels of TMAO have a higher risk of IHD, and the initial circulating level of TMAO and its 10-year change are independently related to subsequent IHD events. This may be related to the gradual increase in TMAO within 10 years, leading to endothelial cell senescence and accelerating vascular senescence. Studies on animal models of accelerated aging mice have found that TMAO can upregulate aging-related β-galactosidase (SA-β-gal) activity. Simultaneously, it promotes increased p53 and p21 expression to accelerate vascular dysfunction and vascular remodeling.

Cell experiments have confirmed that TMAO can inhibit the expression of silencing information regulator 2 related enzyme 1 (SIRT1), increase oxidative stress, reduce endothelial-dependent NO production, and cause endothelial dysfunction. TMAO activates the p53-p21-Rb pathway to induce senescence of human umbilical vein endothelial cells (HUVEC). This may be the mechanism by which TMAO accelerates vascular senescence [65]. A stratified analysis of eating patterns shows that adherence to healthy eating habits may change the unfavorable relationship between TMAO and IHD by adjusting the level of TMAO, and unhealthy eating patterns enhance the TMAO-IHD association. However, healthy eating patterns characterized by higher vegetable intake and lower animal food intake weakened the TMAO-IHD association. This indicates that TMAO can be used as a potential intermediate cut-off point for dietary intervention and that regulating dietary patterns may improve the relationship between TMAO and the incidence of IHD.

In a study of patients with IHD, Zhu et al. [66] found that symptomatic AS is related to changes in human intestinal metagenomics. The abundance of *Enterobacteriaceae*, including *Escherichia coli*, *Klebsiella*, and *Enterobacter aerogenes*, in stool samples of patients with IHD was higher than that in healthy controls. The relative abundance of bacteria such as *Streptococcus* and *Lactobacillus salivarius* present in the oral cavity was also higher than that in the control group. In addition, studies have found that the function of intestinal flora of patients with IHD also undergoes certain changes. Specifically, it exhibits a higher potential to transport monosaccharides and amino acids, whereas the biosynthetic potential of most vitamins is lower. The levels of enzymes that synthesize TMA in the intestinal flora of IHD patients are higher, especially that of YeaW/X. As a homologous enzyme of carnitine monooxygenase (cntA/B), YeaX/X can use carnitine and choline to generate TMA. The increased synthesis of TMAO plays a role in promoting AS somewhat. This indicates that changes in the composition and function of intestinal flora may affect the occurrence and development of IHD to some extent.

### 2.3 Acute coronary syndrome

Gao et al. [67] found that compared with the intestinal flora of patients with healthy controls, the abundance of *Aerococcaceae* and *Eubacterium* was higher, and the abundance of beneficial microbes was lower in the intestinal flora of patients with acute coronary syndrome (ACS). This difference in the composition of intestinal flora is related to serum TMAO levels. Therefore, the specific intestinal flora composition related to serum TMAO levels may also be a potential biomarker for predicting the onset of ACS. Studies have confirmed that the microbial DNA found in AS plaques can also be found in the intestinal tract of the same individual. We hypothesized that intestinal flora is the source of microorganisms in AS plaques and promotes inflammation by producing more pro-inflammatory molecules. This affects the stability of AS plaques and accelerates the progression of CVD.

### 2.4 Myocardial infarction

The inflammatory microenvironment is essential for initiating endogenous repair in the early stages of myocardial infarction (MI) [68]. Recent studies have found that intestinal flora can affect the composition, migration, and function of various immune cell subgroups [69]. As different members of intestinal flora can affect the host's immune homeostasis differently, the heterogeneity of flora may be the basis for individual differences in the host's immune response [70]. In a study of MI mice, Tang et al. [71] found that mice whose intestinal flora was destroyed by antibiotics before MI had a significantly increased, dose-dependent mortality rate after MI. In vivo imaging of the mice showed that the immune activity of the area around the infarction was reduced and that the infiltration of monocytes into the area around the infarction was also reduced, indicating that repair was impaired after MI. Further research found that SCFA are mediated by the homologous SCFA receptor GPR43 or GPR109A and uses DNA methylation to increase the level of plasma L-3,4-dihydroxy-phenylalanine and the abundance of regulatory T (Treg) cells to achieve cardio protection. Dietary supplementation with SCFA can enrich the immune system, restore the immune activity of the area around the infarction, improve the survival rate of mice after MI, and have a protective effect on the development of heart remodeling and myocardial fibrosis. Thus, this new dietary treatment strategy enjoins further research.

The inflammatory response after MI can lead to cardiovascular remodeling and HF, ultimately accelerating the death of the patient. In a study of patients with ST elevation myocardial infarction (STEMI), Zhou et al. [7] found that an increase in intestinal bacterial translocation products (LPS and D-lactate) was associated with increased systemic inflammation and adverse cardiovascular events. D-lactate is a fermentation product of gastrointestinal bacteria. Increases in its blood concentration are due to increases in metabolites produced by intestinal flora in the gastrointestinal tract and in intestinal permeability. Plasma D-lactate levels have been used to assess intestinal damage and are considered sensitive markers for the detection of intestinal barrier damage [72]. Clinical studies have found that plasma levels are negatively correlated with left ventricular ejection fraction and positively correlated with mesenteric artery blood flow [73]. Acute left ventricular dysfunction and low intestinal blood perfusion caused by MI can lead to intestinal barrier dysfunction and increased intestinal mucosal permeability. This leads to the translocation of intestinal flora metabolites into the systemic circulatory system, activating excessive inflammation and increasing the risk of cardiovascular events after MI [74]. Further animal experiments have found that new treatment strategies aimed at protecting the intestinal barrier and inhibiting the translocation of intestinal bacteria may reduce cardiovascular events after MI.

Many clinical studies have shown that elevated TMAO levels are independently associated with an increased risk of MI [8, 75]. In STEMI patients who have undergone secondary preventive treatment, plasma TMAO levels increased slightly from the acute phase to the chronic phase. The TMAO level in the chronic phase evaluated after 10 months is related to the complexity of coronary plaque and the progression of the disease, and it can significantly predict the occurrence of future cardiovascular events in patients with STEMI [76]. The monitoring of TMAO levels is conducive to the timely treatment of MACE in patients with MI. Yang et al. [77] proved that TMAO or a high-choline diet can aggravate cardiac fibrosis in mice after MI. The possible mechanism was the accelerated transformation of fibroblasts into myofibroblasts by activating the TGF-βRI/Smad2 pathway. In that study, it was observed that the plasma TMAO levels of mice in the TMAO or high-choline diet group increased. In addition, the apoptosis and necrotic cell rate after MI, the area of ??myocardial fibrosis in the infarct zone and the distal zone, and the infiltration of macrophages in the infarct zone all showed significant increases. These results indicate that TMAO-aggravated cardiac fibrosis is related not only to the direct activation of fibroblasts but also to enhanced inflammation and promotion of cardiomyocyte apoptosis. Therefore, restricting choline in the diet may help improve the long-term prognosis of patients with MI.

Previous studies have found that PAGln and PAGly have similar functions. They can stimulate the β2 adrenergic receptor (β2AR) to activate the G protein inhibitory α subunit (Gαi)/phosphatidylinositol 3-kinase (PI3K)/protein kinase B (PKB) cascade, which inhibits myocardial cell apoptosis caused by ischemia/reperfusion (I/R) injury [78]. In a mouse I/R injury model, Xu et al. [41] found that continuous administration of appropriate doses of PAGly can inhibit myocardial cell apoptosis caused by myocardial I/R injury in mice and reduce the area of ??MI. However, other studies have found that high-dose PAGly treatment is associated with higher mortality. In animal models of carotid artery injury, elevated levels of PAGln and PAGly led to increased platelet thrombosis in the injured carotid artery. In vitro studies using platelet-rich plasma and isolated platelets have shown that PAGln can enhance the adhesion of platelets to the collagen matrix. PAGln can also dose-dependently enhance the degree of platelet aggregation under the action of a variety of agonists, such as thrombin receptor-activated peptide 6 and collagen, to significantly enhance platelet function, stimulating thrombus formation. The mechanism by which PAGln affects the progression of CVD may be through its combination with ADRs, including α2A, α2B, and β2, to mediate cell response and regulate platelet function and thrombosis potential in vivo [43]. These effects may be related to the dose of PAGln. However, the combined application of PAGly and aspirin can not only reduce the infarct size of I/R injured mice, thereby producing a good therapeutic effect, but also prevent the high mortality caused by high doses of PAGly. This combination of drugs can be used in further clinical research as a new therapeutic strategy for inhibiting myocardial cell apoptosis in patients with MI.

Intestinal flora and its metabolites can affect the development of IHD and the occurrence of MACE after MI by regulating the body's inflammatory response, myocardial fibrosis, and myocardial cell apoptosis. The intervention measures based on the research mentioned here will help improve the long-term prognosis of patients with MI and significantly reduce the mortality rate. The research also suggests that the intestinal flora targeting method has therapeutic potential in reducing the incidence of cardiovascular events after MI.

**Table 1:** The mechanism of intestinal flora and its metabolites in cardiovascular disease.

Diseases	Intestinal flora/metabolite	Effects	Mechanism	Ref.
AS	Bacteroides vulgatus and Bacteroides dorei↓, LPS↑	Damage	Proatherogenic cytokines (IL-2 and 4)↑→ systemic inflammation↑	[55]
AS	LPS↓	Protection	Inflammatory cytokines and chemokines↓	[59]
AS	Intestinal flora (caspase1-/-), SCFA↓	Damage	Inflammatory cytokines (IFN-γ, IL-2 and IL-1β)↑, neutrophil accumulation in atherosclerotic plaques↑	[60]
IHD	TMAO↑	Damage	SA-β-gal activity↑, SIRT1↓→ oxidative stress↑→ p53-p21-Rb pathway↑→ vascular dysfunction and remodeling↑	[64, 65]
IHD	Escherichia coli, Klebsiella, Enterobacter aerogenes, Streptococcus, Lactobacillus salivarius↑	Damage	-	[66]
ACS	Aerococcaceae and Eubacterium↑	Damage	Inflammatory cytokines↑→ plaque stability↓ ?	[67]
MI	SCFA↑	Protection	GPR43/GPR109A↑→ L-3,4-dihydroxyphenylalanine levels and Treg cells↑→ pathological cardiac remodeling↓	[71]
MI	LPS and D-lactate↑	Damage	Systemic inflammation↑	[7]
MI	TMAO↑	Damage	TGF-βRI/Smad2↑→ transformation of fibroblasts into myofibroblasts↑→ cardiac fibrosis↑ Systemic inflammation↑→ cardiomyocyte apoptosis↑	[77]
MI	PAGly/PAGln↑	Protection	β2AR↑→ Gαi/PI3K/PKB↑→cardiomyocyte apoptosis ↓	[41], [78]
	PAGly/PAGln↑	Damage	α2A, α2B, and β2 ADR↑→ platelet responsiveness and thrombosis potential↑	[43]
HF	TMAO↑	Damage	-	[80-82]
HF	Actinobacteria and Bifidobacterium↑	Protection	Ammonia concentration and pH in feces↓	[84]
HF	Shigella, Campylobacter, Yersinia enterocolitica and Salmonella↑	Damage	Pathogenic bacteria↑→ intestinal barrier↓→ chronic systemic inflammation↑	[86]
HF	F.prausnitzii↓, SCFA↓ R. gnavus↑	Damage	Anti-inflammatory effect↓, pro-inflammatory mediators (NO, IL-6, and IL-12)↑→ chronic inflammation↑	[87]
HF/(Cognitive dysfunction)	Gram-negative bacteria containing LPS (Escherichia coli and Shigella etc.) ↑	Damage	Tight junction protein activity ↓ → intestinal barrier↓→ systemic inflammation and neuroinflammation↑ IL-1b and MMP-9↑→ BBB damage↑	[93, 94]
HTN	Prevotella↑ and F.prausnitzii, Bifidobacterium, Coprococcus, Butyrivibrio↓	Damage	Degradation of purine, ketone body biosynthesis and branched-chain amino acid biosynthesis and transport↓, LPS biosynthesis and export, PTS and biosynthesis of phosphatidylethanolamine↑	[96]
HTN	Bacteroides fragilis and arachidonic acid↓	Damage	Intestinal-derived corticosterone production↑→ corticosterone levels in serum↑	[99]
HTN	Odoribacter↑, SCFA↑	Protection	GPR41↑→ systemic blood pressure↓	[100]

(AS= atherosclerosis; LPS= lipopolysaccharide; IL= interleukin; SCFA= short-chain fatty acid; IFN= interferon; IHD= ischemic heart disease;TMAO= trimethylamine-N-oxide; SA-β-gal= aging-related β-galactosidase; SIRT1= silencing information regulator 2 related enzyme 1;ACS= acute coronary syndrome;MI= myocardial infarction;GPR= G-protein-coupled receptor;Treg cells = regulatory T cells; β2AR= β2 adrenergic receptor;Gαi= G protein inhibitory α subunit;PI3K= phosphatidylinositol 3-kinase;PKB= protein kinase B; ADR= adrenergic receptor;HF= heart failure; MMP-9= matrix metalloproteinase-9; BBB= blood-brain barrier;HTN= hypertension; PTS= phosphotransferase system)

### 2.5 Heart failure

HF, which is caused by the failure of the heart to pump enough blood due to pump dysfunction, is the terminal stage of a variety of cardiovascular diseases. It is a disease with a high fatality rate and places a heavy burden on global health and the economy. Unfavorable factors, such as inflammation and immune dysfunction secondary to the imbalance of intestinal flora, have a significant relationship with the occurrence of HF. With bacterial culture methods, more pathogenic bacteria related to chronic inflammation can be found in the stool samples of patients with HF [79].

A study of HF patients found that these patients have increased levels of TMAO and that the predictive value of TMAO for poor prognosis is independent of traditional risk factors and B-type natriuretic peptide levels. Higher TMAO levels are associated with all-cause mortality and poor prognosis in HF patients [80, 81]. Current guideline-based HF treatments do not affect plasma TMAO levels, and patients with higher TMAO levels before and after treatment have a poorer prognosis [82]. Therefore, therapeutic interventions to reduce the level of TMAO may be used as an adjuvant treatment plan for patients with HF. Preliminary clinical trials have found that regulating the composition and function of intestinal flora through diet, use of probiotics, and targeted non-lethal antibacterial enzyme inhibitors can reduce TMAO levels [83]. However, whether these measures will improve the prognosis of patients with HF requires large-scale clinical experimental research.

Hayashi et al. [84] found that *Actinobacteria* and *Bifidobacterium* are enriched in stool samples of HF patients compared with those of the normal group. Moreover, during both the compensatory and the decompensation periods, the plasma TMAO concentration in the HF group is higher than that in the normal group. However, the TMA lyase (*CUTC/D*) gene abundance of the intestinal flora in the decompensated HF phase is significantly higher than that in the compensated HF phase. This suggests that some bacteria containing TMA lyase may be enriched in the decompensated HF phase, thereby affecting the progression of HF. *Bifidobacterium* has a variety of physiological effects, including reduced levels of harmful bacteria, regulation of host immunity, and improvement of the intestinal environment by reducing the concentration of ammonia and pH in feces [85]. Previous studies have found, based on the severity of HF, that HF patients show excessive growth of intestinal pathogens, including *Shigella*, *Campylobacter*, *Yersinia enterocolitica*, and *Salmonella* [86]. For the same patient's stool sample, *Shigella* was more abundant in the decompensated phase of HF than in the compensated phase. This means that,in the process of decompensated HF, there is a vicious cycle of worsening intestinal conditions. Excessive growth of intestinal pathogenic bacteria can damage the intestinal microenvironment and cause intestinal inflammation, leading to the destruction of the intestinal barrier function and systemic inflammation. Furthermore, the decrease in bacterial diversity will negatively affect the nutrition and metabolic efficiency of patients, reduce the production of beneficial metabolites such as SCFA, and accelerate the progression of HF. These findings indicate that the composition of intestinal flora and related metabolites may be related to the pathophysiology of HF, and further research will help identify better diagnostic and treatment methods for HF.

Through the study of stool samples, Cui et al. [87] found that the composition of the intestinal flora of HF patients differed significantly from that of the healthy group, but there was no significant difference in the flora's composition between HF subgroups caused by different etiologies. The decrease in *Faecalibacterium prausnitzii* and the increase in *Ruminococcus gnavus* are the basic characteristics of the intestinal flora of patients with HF.

It is worth mentioning that the *CUTC* gene, which expresses choline TMA lyase, is significantly increased in HF patients, which in turn causes an increase in TMAO levels. At the same time, the abundance of butyrate-acetoacetate CoA transferase in the intestinal flora of HF patients is significantly reduced, resulting in a decrease in the production of protective SCFA butyrate.

Previous studies have confirmed that *F. prausnitzii* is very important for anti-inflammatory activity and maintains the integrity of the intestinal barrier. It can regulate the function of intestinal macrophages and downregulate the levels of pro-inflammatory mediators (such as NO, IL-6, and IL-12) induced by LPS. It can also induce the differentiation of Treg cells and inhibit the inflammatory response and progression of HF. As one of the most abundant butyrate-producing bacteria, *F. prausnitzii* has been identified as an important anti-inflammatory symbiotic bacterium [9]. Its reduction reduces the anti-inflammatory effect and leads to aggravation of chronic inflammation [88]. Studies have found that elderly HF patients have lower levels of *F. prausnitzii* than younger patients, which may be related to the worsening inflammatory state and poor prognosis of elderly HF patients [89], whereas the level of *R. gnavus* increases significantly in patients with HF. Previous studies on inflammatory bowel disease have shown that *R. gnavus* has pro-inflammatory properties. After implantation in sterile mice, the levels of IFN-γ and IL-17 in blood were significantly increased, leading to the activation of the body's inflammatory response [90]. Thus, HF ??is related to different intestinal flora disorders. For some specific core bacterial imbalances, follow-up studies are needed to confirm whether there is a causal relationship and to explore related intervention strategies.

Cognitive impairment is a common central nervous system complication after HF, with an incidence rate of 25% to 75% [91]. The main mechanisms include low cerebral blood perfusion, abnormal hormone secretion, and an inflammatory response [92]. Increasing evidence shows that there is also a relationship between intestinal flora imbalance and neuroinflammation. In an ischemic HF rat model study, Yu et al. [93] found that HF promotes the growth of LPS-containing gram-negative bacteria, including *E.coli and Shigella*. This leads to an imbalance of intestinal flora, inhibits the activity of tight junction proteins, and destroys the intestinal barrier. This phenomenon causes pathogenic bacteria expressing LPS in the intestine to escape, activate immune cells, and trigger an inflammatory response, leading to systemic inflammation and neuroinflammation. Further animal studies have confirmed that the levels of IL-1b and matrix metalloproteinase-9 (MMP-9) in HF rats were elevated. IL-1b can upregulate the expression of MMP-9 mRNA and protein, leading to increased degradation of basal components by MMP-9, destroying the tight junctions of the blood-brain barrier (BBB), and increasing the exposure of brain tissue to various cytokines. This exposure eventually leads to cognitive impairment [94]. The intestinal flora imbalance caused by HF aggravates neuroinflammation in part by impairing the permeability of the BBB and intestinal barrier. Further studies have found that the intake of probiotics can affect the synthesis of pro-inflammatory cytokines by inhibiting the imbalance of intestinal flora, playing a role in improving cognitive impairment. These findings may not only explain the underlying mechanism of cognitive dysfunction observed after HF but also promote in-depth exploration of the treatment of cognitive dysfunction caused by HF by improving intestinal flora.

The composition of intestinal flora and its metabolites not only is related to the disease progression of HF patients but also affects the long-term prognosis of HF directly or indirectly, leading to various complications. Treatment options for these problems may include optimizing the composition of intestinal flora, inhibiting translocation of intestinal flora, and reducing the level of related metabolites; however, further research is needed.

### 2.6 Hypertension

HTN is a multifactorial disease that depends on the complex interactions between genetic and environmental factors. Previous studies have found that, compared with conventional mice, sterile mice without intestinal bacteria exhibit lower blood pressure [95]. Therefore, we believe that there may be a connection between intestinal flora and HTN. In a large clinical study of patients with HTN [96], *Prevotella* increased significantly in the HTN group, and *F. prausnitzii*, *Bifidobacterium*, *Coprococcus*, and *Butyrivibrio*, which were enriched in healthy controls, decreased in the HTN group. The decreased level of *F. prausnitzii* in the intestine is related to inflammatory bowel disease and is essential for the production of butyrate. *Bifidobacterium* is necessary to maintain homeostasis of intestinal flora and protect the intestinal barrier. The overgrowth of harmful flora and the decrease in beneficial flora, accompanied by adverse changes in the function of intestinal flora, play an important role in the pathological process of HTN. The function of the bacteria associated with HTN in the degradation of purine nucleotides, the biosynthesis of ketone bodies, and the synthesis and transportation of branched-chain amino acids is reduced. However, the function of the bacteria associated with HTN is significantly improved with respect to the biosynthesis and transport of LPS, an important mechanism of inflammation that leads to the progression of intestinal tumors [97], and to the phosphotransferase system related to diabetes, liver cirrhosis, rheumatoid arthritis [98], and biosynthesis of phosphatidylethanolamine.

Studies have found that a high-salt diet can cause HTN because of the decrease in *Bacteroides fragilis* in the intestine and a decrease in the content of its metabolite arachidonic acid. This increases the production of intestinal corticosterone and the level of corticosterone in the intestines and serum, leading to an increase in blood pressure. The HTN caused by this high-salt content can be transferred through fecal microbiota transplantation, indicating the key role of intestinal microbiota in developing the disease [99]. Studies have confirmed that the abundance of butyrate-producing bacteria, *Odoribacter*, in the intestines of obese pregnant women is negatively correlated with systolic blood pressure (SBP). Furthermore, in vitro experiments have confirmed that SCFA can reduce systemic blood pressure by activating GPR41 [100]. Therefore, supplementation of butyrate-producing bacteria in the intestine may help obese pregnant women maintain normal blood pressure. There are broad research prospects for the relationship between HTN and intestinal flora, but an in-depth study of the pathophysiological mechanism depends on further exploration (Table 1).

## 3. The therapeutic intervention of intestinal flora

Intestinal flora is closely related to cardiovascular health and disease. In the treatment of CVD, researchers have gradually turned their attention to intestinal flora and related metabolites. As a newly identified regulator of CVD, the gut microbiome has become a potential target for treatment (Table 2).

### 3.1 Individualized diet intervention

There is a mutually beneficial relationship between the diet and intestinal microbiota. Therefore, diet is an important external factor affecting the intestinal microbiota and is the most effective regulator of its composition and function. The risk of CVD can be effectively reduced by improving diet or supplementing nutrients [101, 102].

**Table 2:** The therapeutic intervention of intestinal flora in cardiovascular diseases.

Therapeutic intervention	Intestinal flora/metabolite	Mechanism	Ref.
Diet
Vegetarian diet	Ruminococcus and Pasteurella↑	Oxidized LDL-C↓	[103]
Whole-grain diet	Lachnospira↑and Enterobacteriaceae↓	-	[106]
Lower-fat diet	Akkermansia muciniphila and F.prausnitzii↑, SCFA↑	Palmitic acid, stearic acid, indole, hs-CRP and thromboxane B2↓	[107], [110]
Rich in nuts diet	F.prausnitzii and Roseburia↑, Ruminococcus and Dorea↓	Proinflammatory secondary bile acids and LDL-C↓	[114]
Rich in propionate diet	Bifidobacterium↑	Insulin sensitivity↑, Treg cells↑and Th17 cells↓→ systemic inflammation↓	[117]
Soy	Prevotella and Dialister↓	-	[118]
Fecal microbiota transplantation			
Control → high-calorie diet mice	-	Disrupted glucose metabolism↓	[126]
Control → germ-free mice	-	Arterial remodeling response↑	[127]
WKYs→ SHRs	-	Th17 cells/Treg cells in MLNs and aorta↓→ endothelial dysfunction↓	[128]
Control → EAM	Bacteroidetes↑→F/B↓	IFN-γ gene expression↓→ inflammatory infiltration↓→myocardialdamage↓	[129]
Probiotic			
Lactobacillus reuteri	-	TC↓, HDL-C↑	[130]
Lactobacillus acidophilus, Bifidobacterium lactis and Lactobacillus salivarius	-	PWV↓, IL-6, TNF-α and TM↓	[131]
Lactobacillus acidophilus and Bifidobacterium lactis	-	Insulin sensitivity↑, TC/HDL-C and triglycerides↓, HDL-C↑ PAI-1 and VCAM-1↓	[134-136]
Lactobacillus plantarum, Lactobacillus acidophilus and Lactobacillus reuteri	-	Hs-CRP and TNF-α↓, lipid metabolism↑	[137]
Lactobacillus plantarum	-	IL-8, IL-12 and leptin↓	[138]
Probiotics with high biliary salt hydrolase activity(Bifidobacterium longum, Lactobacillus plantarum etc.)	-	Triglycerides and LDL-C↓	[139, 140]
Bifidobacterium animalis subsp. lactis 420	SCFA↑	Glycoursodeoxycholic acid and tauroursodeoxycholic acid↓	[141]
Bifidobacterium and arginine	-	Spermidine↑→autophagy↑	[142]
Lactobacillus acidophilus, Lactobacillus casei and Bifidobacterium bifidum	-	Hs-CRP and MDA↓, NO↑	[143]

(LDL-C= low-density lipoprotein cholesterol; SCFA= short-chain fatty acid; hs-CRP= high-sensitivity C-reactive protein; Treg cells = regulatory T cells; Th17 cells = T helper 17 cells; WKYs= Wistar-Kyoto rats; SHRs= spontaneously hypertensive rats; MLNs= mesenteric lymph nodes; EAM= experimental autoimmune myocarditis; F/B= Firmicutes to Bacteroidetes; IFN= interferon; TC= total cholesterol; HDL-C= high-density lipoprotein cholesterol; PWV= pulse wave velocity; IL= interleukin; TNF= tumor necrosis factor; TM= thrombomodulin; PAI-1= plasminogen activator inhibitor-1; VCAM-1= vascular cell adhesion molecule-1; MDA= malondialdehyde)

Changes in diet, including vegetarian, whole-grain, and lower-fat diets, have significant preventive effects on CVD. Isocaloric vegetarian food can significantly reduce plasma total cholesterol (TC), oxidized low-density lipoprotein cholesterol (LDL-C) levels, and body mass index compared with isocaloric meat [103]. Among the individuals with greater reduction in oxidized LDL-C, *Ruminococcus* and *Pasteurella* were more abundant. This suggests the interaction between a specific intestinal flora composition and a vegetarian diet can reduce the level of oxidized LDL-C. Elevated oxidized LDL-C can activate the innate immunity and adaptive immune system, leading to an increase in the release of AS-related inflammatory factors. It is a powerful predictor of future IHD events independent of traditional cardiovascular risk factors [104, 105]. Whole-grain diets can promote the growth of beneficial bacteria and limit the growth of known opportunistic pathogens. Specifically, the SCFA producer *Lachnospira* increased, the pro-inflammatory *Enterobacteriaceae* decreased [106]. Additionally, a low-fat diet combined with lifestyle intervention alleviated the dysbiosis of the gut microbiota [107], which specifically manifests with an increase in *F. prausnitzii* and *Akkermansia muciniphila*.

*F. prausnitzii* has been identified as an important anti-inflammatory symbiotic bacterium. A high level of *A. muciniphila* is positively correlated with insulin sensitivity, and the specialization of A. muciniphila in the degradation of mucin makes it a key microorganism for maintaining intestinal barrier function [108, 109]. Furthermore, a low-fat diet can increase the level of SCFA in feces; reduce the concentration of indole, palmitic acid, and stearic acid; and reduce the level of LPS in the blood. Moreover, it can reduce the level of inflammatory factors, including high-sensitivity C-reactive protein (hs-CRP) and thromboxane B2, in the plasma, reducing the risk of AS [110]. Indole is the precursor of indoxyl sulfate, which is related to HTN and CVD in patients with chronic kidney disease [111]. Palmitic acid and stearic acid are the main saturated fatty acids in food and tissues and can stimulate the inflammatory signal transduction of macrophages and fat cells. Epidemiological studies have confirmed that palmitic acid and stearic acid are positively correlated with CVD [112, 113]. These changes in diet can prevent or treat CVD by improving the imbalance of intestinal flora.

In addition, dietary intervention also includes supplementation with some nutrients to better regulate intestinal flora and plays a role in disease treatment. After eating nuts, patients showed increased *F. prausnitzii* and *Roseburia* content, decreased *Ruminococcus* and *Dorea* content, and reduced secondary bile acid levels in their feces [114]. The significant reduction in microbial-derived pro-inflammatory secondary bile acids, such as deoxycholic acid and lithocholic acid, has been shown to reduce intestinal inflammation [115, 116]. Normally, *Dorea* and *Roseburia* are related to the concentration of secondary bile acids. This suggests that the relationship between the gut microbiota and CVD may be related to the microbial metabolism of bile acids. Selectively increasing the level of propionate in the diet can regulate the body's amino acid metabolism, improve insulin sensitivity, increase the proportion of Treg cells, reduce the level of pro-inflammatory T helper 17 (Th17) cells, and exert anti-inflammatory effects. It can also promote the growth of *Bifidobacterium* to play a protective role in the intestinal tract [117]. Interestingly, soy consumption can reduce the systolic blood pressure of patients with HTN by inhibiting specific intestinal flora such as *Prevotella* and *Dialister.*However, this only works if the gut microbiota is sensitive to soy [118]. This is worth further study as a potential treatment.

### 3.2 Fecal microbiota transplantation

Fecal microbiota transplantation (FMT) is defined as the transfer of microbial communities from healthy donors to the patient's intestines. FMT is an effective, cheap, and safe method that is clinically used to enrich human intestinal flora to treat certain chronic diseases, especially recurrent *Clostridium difficile* infection [119-121]. In recent years, studies have found that FMT also has potential therapeutic value in many aspects, such as type 2 diabetes, irritable bowel syndrome, inflammatory bowel disease, metabolic syndrome (MetS), and CVD [122].

MetS is a complex multifactorial disease. Its components include obesity, dyslipidemia, glucose intolerance, and HTN, which can increase the risk of CVD, type 2 diabetes, and stroke [123]. The results of a meta-analysis showed that MetS doubled the risk of CVD and increased all-cause mortality by a factor of 1.5. However, there is still a lack of effective drugs for treatment [124]. Studies have found that FMT from vegan donors can cause detectable changes in the composition of the gut microbiota in patients with MetS but cannot cause changes in TMAO production capacity or parameters related to vascular inflammation [125]. A high-calorie diet induced glucose intolerance and obesity in mice. FMT from normal mouse donors can alleviate this glucose intolerance in recipient mice, showing potential therapeutic value [126]. Compared with that in conventionally reared mice, neointimal hyperplasia in sterile mice is significantly weakened after carotid artery ligation, but the arterial intimal hyperplasia can be restored by using conventionally reared mice as donor FMT. This phenomenon may be related to the regulation of local arterial inflammation by microbiota metabolites [127].

In rats with normal (Wistar-Kyoto rats, WKYs) to spontaneously hypertensive (SHRs) blood pressure, FMT can reduce the basal SBP of SHRs, the aorta and mesenteric lymph nodes (MLNs), the ratio of pro-inflammatory/anti-inflammatory cells (Th17 cells/Treg cells), and the endothelial dysfunction and vascular oxidation state of SHRs. After FMT from SHRs to WKYs, the levels of CD80 and CD86 mRNA in MLNs were found to be increased, the endothelial function was impaired, and the underlying SBP increased. Further studies have found that this impaired endothelial function caused by the imbalance of intestinal flora may be mediated by increased IL-17 production. Using IL-17 inhibitors can lower the blood pressure of SHRs and restore endothelial function. This indicates that the Th17/IL-17 axis is essential for the development of endothelial dysfunction and HTN caused by FMT from SHRs to WKYs [128].

Transplanting the fecal contents of normal mouse donors into the intestine of experimental autoimmune myocarditis (EAM) mice can reduce the expression of the IFN-γ gene in heart tissue, inflammation infiltration, and myocardial damage. Furthermore, studies have confirmed that myocarditis is related to the imbalance of the intestinal microbiota, which is characterized by an increased ratio of *Firmicutes* to *Bacteroidetes* (F/B). FMT can reduce F/B by restoring the abundance of *Bacteroidetes* and the balance of intestinal flora; therefore, FMT may be a potential treatment strategy for myocarditis [129]. Although evidence suggests that FMT is a safe treatment with few side effects, the long-term results of FMT have not yet been fully elucidated. Therefore, regular follow-up after FMT to monitor clinical efficacy and long-term adverse events is an important matter for debate.

### 3.3 Probiotics

Probiotics are microorganisms that are beneficial to health. Their therapeutic potential has led to a significant increase in research interest to regulate gut microbiota to treat CVD in recent years.

*Lactobacillus reuteri* reduced plasma TC levels and increased high-density lipoprotein cholesterol (HDL-C) levels in rats fed a high-fat diet [130]. Supplementing multi-species probiotics, including *Lactobacillus acidophilus*, *Bifidobacterium lactis*, and *L. salivarius*, to menopausal obese women [131] can reduce the blood levels of IL-6, TNF-α, and thrombomodulin, one of the most sensitive and specific markers of endothelial damage [132]. Furthermore, it can significantly reduce SBP and pulse wave velocity (PWV) [133]. PWV is the most accurate method for non-invasive estimation of human arterial stiffness. The combination of *L. acidophilus* and *B. lactis* can also significantly reduce fasting blood glucose and serum insulin concentrations in patients with diabetic nephropathy [134] and diabetes with IHD [135], increase insulin sensitivity, reduce triglyceride levels and TC/HDL-C ratio, and increase HDL-C levels. In addition, in patients with MetS, the combination of the two can reduce the plasma levels of plasminogen activator inhibitor-1 and vascular cell adhesion molecule-1 (VCAM-1), both of which are vascular inflammation markers of endothelial dysfunction. Epidemiological studies have found a significant positive correlation between high levels of VCAM-1 and CVD [136].

The combination of *Lactobacillus plantarum*, *L. acidophilus*, and *L. reuteri* can reduce serum hs-CRP and TNF-α levels in elderly patients with MetS to exert anti-inflammatory effects [137]. Furthermore, it can improve blood lipid metabolism and has potential therapeutic value for CVD. Moreover, supplementing *L. plantarum* in patients with IHD can reduce the circulating levels of IL-8, IL-12, and leptin and reduce systemic inflammation independent of traditional risk factors and changes in TMAO levels [138]. Probiotics with high bile salt hydrolase activity, such as *Bifidobacterium longum* [139] and *L. plantarum*[140], can reduce triglycerides and LDL-C by reducing intestinal cholesterol reabsorption, helping to control high cholesterol in patients with CVD. Supplementation with Bifidobacterium animalis ssp. lactis 420 in the diet of obese patients can reduce the levels of glycoursodeoxycholic acid and tauroursodeoxycholic acid in plasma while increasing SCFA levels to exert anti-inflammatory protective effects [141].

Spermidine-induced autophagy has been shown to reduce the risk of CVD in mice, and the combination of *Bifidobacterium* and arginine can reduce the risk of AS by increasing blood spermidine levels [142]. In the blood of patients with IHD, the combination of *L. acidophilus*, *Lactobacillus casei*, and *Bifidobacterium bifidum* can significantly reduce the levels of hs-CRP and malondialdehyde (MDA) and increase the level of nitric oxide [143]. Existing studies have found that reducing hs-CRP and increasing nitric oxide levels can reduce vascular oxidative stress, endothelial dysfunction, and CVD events [144]. MDA is the most important marker of membrane lipid peroxidation caused by reactive oxygen species. It can indirectly reflect the level of oxidative stress [145] and is related to many diseases, including cancer, CVD, and type 2 diabetes [146]. The above results show that the reasonable application of probiotics may provide potential treatment options for patients with dyslipidemia, diabetes, and CVD. However, the intake of probiotics can only be a potential supplement to more traditional cardiovascular treatments and non-drug measures used to prevent disease progression. To determine the safety and effectiveness of this treatment, more research is needed.

## 4. The latest developments regarding intestinal flora in clinical applications

Up to now, there have been many clinical studies on the prevention and treatment of CVD by regulating the intestinal flora and its metabolites. MetS, as one of the risk factors of CVD, includes a series of metabolic diseases, such as hypertriglyceridemia, insulin resistance, and impaired glucose tolerance. Central obesity and insulin resistance are currently recognized as important pathogenic factors for MetS [147]. In a clinical study of 29 overweight individuals, Vitae et al. [148] found that, compared with the conventional Western diet, the Mediterranean diet can reduce oral glucose insulin sensitivity and LDL-C levels to a greater extent. It also results in decreases in the abundance of *Ruminococcus torques*, *Coprococcus comes*, *Streptococcus gallolyticus*, and *Flavonifractor plautii* and increases in the abundance of *Intestinimonas butyriciproducens* and *Akkermansia muciniphila*. In a randomized clinical trial of 39 patients with MetS, Guo et al. found that intermittent fasting may play a cardiovascular protective role by increasing the production of SCFA and reducing the level of LPS in the circulatory system [149]. Depommier et al. found in a study of 40 overweight patients with insulin resistance that dietary supplementation of *Akkermansia muciniphila* can reduce gamma-glutamyl transferase, aspartate-aminotransferase, and other liver transaminase levels [150], which are associated with adverse changes in glucose and lipid metabolism. At the same time, such supplementation reduces the level of LPS in the blood, which has a potential preventive effect on CVD. In a study of 78 individuals with central obesity [151], eating foods rich in polyphenols and long-chain n-3 polyunsaturated fatty acids was shown to significantly increase the levels of *Bifidobacteria* and *Ruminococcaceae*, reduce the abundance of Lachnospiraceae and improve insulin secretion and blood lipid levels, thereby reducing the risk of morbidity in people with high CVD risk. Thus, we can speculate that the gut microbiota may be involved in improving glucose metabolism and insulin sensitivity, thereby affecting the progression of CVD. Chen et al. studied 145 patients with untreated HTN and found that a moderate reduction in dietary sodium content increases circulating SCFA levels [53], which is associated with reduced BP. Supporting dietary sodium may affect the progression of CVD by affecting the gut microbiota.

**Figure1.**
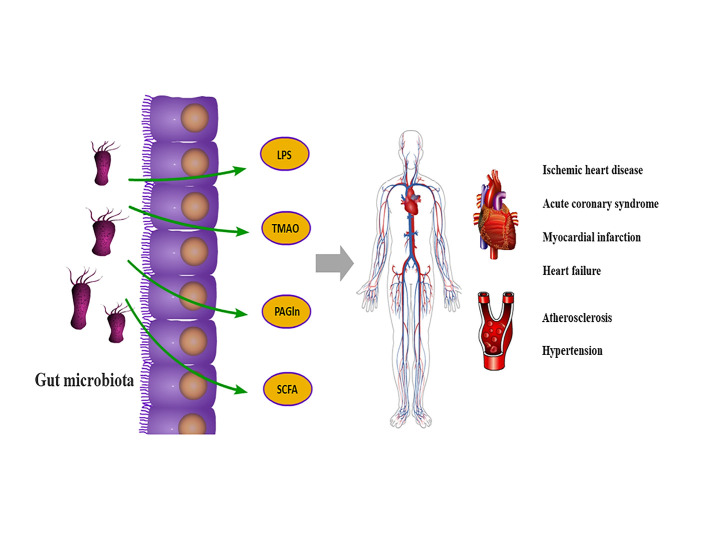
The role of intestinal microbiota in cardiovascular diseases.

The research on the role of intestinal flora and its metabolites in the diagnosis and treatment of CVD is still in the clinical trial stage and has not been widely used in clinical practice. However, these latest studies show that the supplement of intestinal flora to traditional disease diagnosis and treatment programs is huge and exciting. We have reason to believe that, in the future, the intestinal flora, as a brand-new regulator, will play a greater role in reducing the global prevalence of CVD and CVD-related mortality and improving the average life expectancy of the global aging population (Fig. 1).

## 5. Conclusion

Current research shows there is an important link between intestinal flora and cardiovascular disease, emphasizing that intestinal flora and its metabolites are closely related to diseases such as ischemic heart disease, heart failure, and hypertension. However, the existing evidence mainly includes small-sample clinical studies and cross-sectional studies with inconsistent results. The most important pathophysiological link between intestinal flora and cardiovascular disease seems to be the induction of the host inflammatory response through changes in the composition or function of the flora. However, whether this change has a clear causal relationship with cardiovascular disease and whether it is affected by other factors remain to be confirmed. This article also points out the potential therapeutic value of intestinal flora for cardiovascular disease, including personalized diet intervention, intestinal flora transplantation, and the rational application of probiotics. These findings provide the potential for the development of new strategies for the prevention and treatment of cardiovascular disease by targeting intestinal flora.
